# NPC1 Deficiency in Mice is Associated with Fetal Growth Restriction, Neonatal Lethality and Abnormal Lung Pathology

**DOI:** 10.3390/jcm9010012

**Published:** 2019-12-19

**Authors:** Jorge L. Rodriguez-Gil, Dawn E. Watkins-Chow, Laura L. Baxter, Tadafumi Yokoyama, Patricia M. Zerfas, Matthew F. Starost, William A. Gahl, May Christine V. Malicdan, Forbes D. Porter, Frances M. Platt, William J. Pavan

**Affiliations:** 1 Genomics, Development and Disease Section, Genetic Disease Research Branch, NHGRI, NIH, Bethesda, MD 20892, USA; jorge.rodriguez-gil@nih.gov (J.L.R.-G.); dchow@mail.nih.gov (D.E.W.-C.); lbaxter@mail.nih.gov (L.L.B.); 2 Department of Pharmacology, University of Oxford, Oxford OX1 3QT, UK; 3 Medical Scientist Training Program, University of Wisconsin-Madison School of Medicine and Public Health, Madison, WI 53726, USA; 4 Section of Human Biochemical Genetics, Medical Genetics Branch, National Human Genome Research Institute, NHGRI, NIH, Bethesda, MD 20892, USA; tadafumiy@staff.kanazawa-u.ac.jp (T.Y.); gahlw@mail.nih.gov (W.A.G.); maychristine.malicdan@nih.gov (M.C.V.M.); 5 Office of Research Services, Division of Veterinary Resources, NIH, Bethesda, MD 20892, USA; zerfasp@ors.od.nih.gov (P.M.Z.); starostm@ors.od.nih.gov (M.F.S.); 6 Program in Developmental Endocrinology and Genetics, NICHD, NIH, Bethesda, MD 20892, USA; fdporter@mail.nih.gov

**Keywords:** Lysosomal storage diseases, NPC1, Niemann-Pick disease type C, neonatal lethality, lung surfactant

## Abstract

The rare lysosomal storage disorder Niemann-Pick disease type C1 (NPC1) arises from mutation of *NPC1*, which encodes a lysosomal transmembrane protein essential for normal transport and trafficking of cholesterol and sphingolipids. NPC1 is highly heterogeneous in both clinical phenotypes and age of onset. Previous studies have reported sub-Mendelian survival rates for mice homozygous for various *Npc1* mutant alleles but have not studied the potential mechanisms underlying this phenotype. We performed the first developmental analysis of a *Npc1* mouse model, *Npc1^em1Pav^*, and discovered significant fetal growth restriction in homozygous mutants beginning at E16.5. *Npc1^em1Pav/em1Pav^* mice also exhibited cyanosis, increased respiratory effort, and over 50% lethality at birth. Analysis of neonatal lung tissues revealed lipid accumulation, notable abnormalities in surfactant, and enlarged alveolar macrophages, suggesting that lung abnormalities may be associated with neonatal lethality in *Npc1^em1Pav/em1Pav^* mice. The phenotypic severity of the *Npc1^em1Pav^* model facilitated this first analysis of perinatal lethality and lung pathology in an NPC1 model organism, and this model may serve as a useful resource for developing treatments for respiratory complications seen in NPC1 patients.

## 1. Introduction

Niemann-Pick disease, type C (NPC) is a rare, autosomal recessive, lysosomal storage disorder affecting approximately 1 in 120,000 to 150,000 live births [1,2]. The majority (95%) of causative mutations underlying NPC have been identified in *NPC1* (NPC1 disease, OMIM #257220), while the remaining 5% are located in *NPC2* (NPC2 disease, OMIM #607625) [1,3,4]. Both *NPC1* and *NPC2* encode lysosomal proteins that regulate cholesterol transport and trafficking. Loss of function mutations in either *NPC1* or *NPC2* result in the accumulation of unesterified cholesterol, sphingomyelin and other sphingolipids within the late endosome/lysosome [5,6,7]. NPC1 is a highly conserved transmembrane protein with a sterol-sensing-domain that shares sequence homology with other sterol-sensing proteins, including the Hedgehog signaling protein receptor Patched [3,7,8,9,10,11,12]. NPC1 patients exhibit cellular accumulation of cholesterol and sphingolipids which leads to visceral phenotypes of splenomegaly or hepatomegaly, along with a broad range of neurological phenotypes that include cerebellar-related ataxia [13,14].

NPC1 disease has an extremely variable clinical presentation, including an age of onset that ranges from in utero to adulthood [14,15,16]. The current NPC1 literature primarily describes the most commonly occurring infantile and juvenile ages of onset, with very few studies focused on pre/perinatal cases with an age of onset before 2 months [14,16,17,18,19,20,21,22]. NPC1 patients suffering from this earlier presentation are characterized by more notable visceral clinical findings, such as splenomegaly and/or hepatomegaly as well as ascites (summarized in [22]); in contrast, later onset patients manifest the classical neurodegenerative symptoms of NPC1 [14]. Based on the few described cases, the outcome for pre/perinatal NPC1 is very poor, and many patients reportedly die from cholestatic liver disease within the first few weeks of life [16]. However, several recent reports have described respiratory complications as the main cause of mortality in neonatal NPC1 patients [23,24]. 

Several clinical trials utilizing multiple treatment approaches for NPC1 disease are currently being performed [2]. While the majority of all human trials are based on results derived from preclinical studies using animal models [25], one can argue that model organisms play an even more vital role in understanding NPC1. Since NPC1 is a rare disease, sample size is greatly reduced and often considered a limiting factor in clinical trial design [26]. Moreover, the genetic and clinical heterogeneity seen in NPC1 disease requires varied animal models to reproduce these differences and to provide relevant models to assess targeted treatments that are tailored to specific types of mutations [27,28,29]. While current models recapitulate features seen in infantile and adult onset NPC1 patients [29], additional studies are needed to identify animal models that exhibit the more severe pre/perinatal presentation of NPC1. Of note, it has been reported that multiple *Npc1* mouse models show a sub-Mendelian ratio of homozygous mutant/null animals after genotyping, ranging from 2.8% to 18% [30,31]. However, the fundamental question of when these affected mice are dying—in utero, at birth, or in the postnatal period prior to genotyping—remains unanswered. In this study, we present the first developmental analysis of a *Npc1* mouse model as well as detailed characterization of a neonatal lethality phenotype associated with novel abnormal lung pathology in a mouse model of NPC1, using the recently described *Npc1^em1Pav^* mouse model (hereafter abbreviated *Npc1^em^*, Rodriguez-Gil et al., under revision). These results suggest a potentially unifying explanation for the sub-Mendelian ratio observed in multiple *Npc1* mutant mouse strains and may have implications for treatment/diagnosis of pre/perinatal NPC1 patients.

## 2. Experimental Section

### 2.1. Mouse Genetic Strains, Mouse Genotyping and Maintenance 

All mouse colonies (protocol number ASP: G-94-7, approved on 12 November, 2017) were maintained in a specific pathogen-free (SPF), AAALAC-approved facility in accordance with National Institutes of Health (NIH) guidelines and following the standards required by the NHGRI Animal Care and Use Committee (ACUC). All mice used in this sudy were maintained on a standard Purina Prolab RMH 1800 diet. The *Npc1^em1Pav^* line (abbreviated *Npc1^em^*) was previously generated by CRISPR/Cas9 targeting of exon 21 of *Npc1* on a C57BL/6J background, which caused a nine-base pair, in-frame deletion that is predicted to result in deletion of 3 amino acids, Ser1062, Asn1063, and Ile1064 (Rodriguez-Gil et al., under revision). Analyses of *Npc1^em/em^* mutants showed that these mice had significantly reduced NPC1 protein levels and displayed the pathological and biochemical hallmarks of NPC1 disease (Rodriguez-Gil et al., under revision). The colony was maintained by backcrossing to C57BL/6J (Jackson Laboratories Stock #000664) and heterozygotes were mated to generate homozygote mice for analysis. An end-point real-time PCR assay was used for genotyping. Samples were amplified with the following probes and primers using a Universal 2x Taqman Master Mix (Thermo Fisher Scientific) and an ABI 7500 instrument for thermocycling and detection: Mutant, FAM-TTACTGGCTGTTAGCCG-MGBNFQ; WT, VIC-ATGTTACTGGCTATTAGCCG- MGBNFQ; F, GCGGTAGTCACTCCCCTTAG; R, CCATGAAGAAAGCTCGGCTA. 

B6.C-*Npc1^m1N^*/GarvJ mice, which carry a null mutation for *Npc1* [3], were imported directly from the laboratory of Dr. William S. Garver at the University of New Mexico (currently available at Jackson Laboratories, stock #030097). Prior to import, these mice had been backcrossed from their original strain (BALB/cNctr) to C57BL/6J for over thirty generations (Dr. William S. Garver, personal communication). B6.129-*Npc1^tm1Dso^*/J mice, which carry a targeted gene replacement at *Npc1* that mimics the common I1061T human *NPC1* mutation [32], were imported directly from Washington University School of Medicine in St. Louis from the laboratory of Dr. Daniel S. Ory (currently available at Jackson Laboratories, stock #027704). Marker-assisted selection (Speed Congenics) was used to quickly establish *Npc1^em^* mutants with a greater percentage of BALB/cJ homozygosity (currently available at Jackson Laboratories, stock #000651). Mouse genomes were assessed at the DartMouse^TM^ Speed Congenics Core Facility (Geisel School of Medicine, Darthmouse College) using a high-density SNP-array. Subsequent backcrosses were performed for 6 generations, and these mice showed homozygosity for BALB/cJ at >92% of markers at approximately 3000 strain-specific SNPs. 

### 2.2. Organ Weights and Dissections 

Dissections were performed under a stereomicroscope (Carl Zeiss AG Discovery V12, Carl Zeiss Inc., Thornwood, NY, USA) to remove organs after cesarean section. Briefly, a midline incision was made into the abdomen in order to expose the xiphoid process. The thoracic cavity was exposed and the thymus, heart, trachea and all lung lobes were removed en bloc. Each organ was immersed in 1X phosphate-buffered saline (PBS) and dissected using forceps. All organs were weighed using a digital scale (Sartorius, CPA225D Semimicro Balance, Sartorius AG, Goettingen, Germany). 

### 2.3. Embryo and Tissue Histology/Staining

All embryos and tissues were harvested and fixed overnight at 4 °C in 4% paraformaldehyde (Ted Pella Inc., Redding, CA, USA). Tissues were then washed twice with 1X PBS before storage in 70% ethanol at 4 °C until histology processing (Histoserv Inc., Germantown, MD, USA). Briefly, samples were dehydrated through graded alcohols, cleared in xylene, and infiltrated with paraffin. After processing, all tissues were embedded in paraffin and cut into 5 μm sections on a microtome and stained with hematoxylin and eosin. For Masson’s trichrome staining, samples were left in Bouin’s solution overnight. They were then rinsed thoroughly and transferred to Weigert’s hematoxylin. After hematoxylin, the slides were rinsed in water then placed in Biebrich scarlet-acid fuchsin solution. The slides were rinsed again in water, before transferring to phosphomolybdic-phosphotungstic acid solution. Slides were rinsed in water and put in aniline blue, rinsed in water a final time, and then dehydrated through graded alcohols and placed in xylene. In addition to gross observations, assessment of morphology and organogenesis in embryos included histological analysis of liver, spleen, thorax, and placenta.

### 2.4. Cesarean Delivery and Fostering Experiments 

Timed matings were set up by crossing heterozygote *Npc1^em/+^* females with *Npc1^em/+^* males. The identification of a vaginal plug was considered as half an embryonic day (E0.5). All females were separated and housed individually after a vaginal plug was identified. Pregnant females were injected subcutaneously with 50 mg/ml pharmaceutical-grade medroxyprogesterone acetate (Depo-Provera, Pfizer, NY, USA) at E16.5 and E18.5 in order to prevent parturition, as previously described [33]. 

A non-recovery cesarean section was performed at E19.5. Briefly, embryos were carefully removed from the uterine tube using blunt forceps. The placenta was also removed, and umbilical cords were cut. Newborn pups were placed on a warmed surface (37°C) and massaged with a clean Q-tip soaked in warmed PBS. Pups were introduced to foster FVB/NJ mothers (Jackson Laboratories) with biological newborns between 1 and 2 days old with litter size not exceeding 10 pups. Newborn pups were monitored regularly for the first ten days after birth. Fostered pups were immediately recognizable due to the black coat color of the *Npc1^em^* background strain (C57BL/6J) compared to albino FVB/NJ pups. 

### 2.5. Transmission Electron Microscopy 

Lung tissues (1 mm^3^) were taken immediately following C-section and fixed for 48 h at 4 °C in 0.1M cacodylate buffer (pH 7.4) containing 2% glutaraldehyde and 1% paraformaldehyde before being washed with cacodylate buffer three times. Tissues were then fixed with 1% OsO_4_ for two hours, washed with 0.1M cacodylate buffer three times, washed with water, and placed in 1% uranyl acetate for one hour at room temperature. Samples were subsequently serially dehydrated in ethanol and propylene oxide and embedded in EMBed 812 resin (Electron Microscopy Sciences, Hatfield, PA, USA). Thin sections, approximately 80 nm, were obtained by utilizing the Leica ultracut-UCT ultramicrotome (Leica, Deerfield, IL, USA) and placed onto 300 mesh copper grids and stained with saturated uranyl acetate in 50% methanol and then with lead citrate. The grids were viewed in a JEM-1200EXII electron microscope (JEOL Ltd, Tokyo, Japan) at 80 kV and images were recorded with a XR611M, mid-mounted, 10.5 M pixel, CCD camera (Advanced Microscopy Techniques Corp, Danvers, MA, USA). 

### 2.6. Statistical Analysis

**Figure 1:**
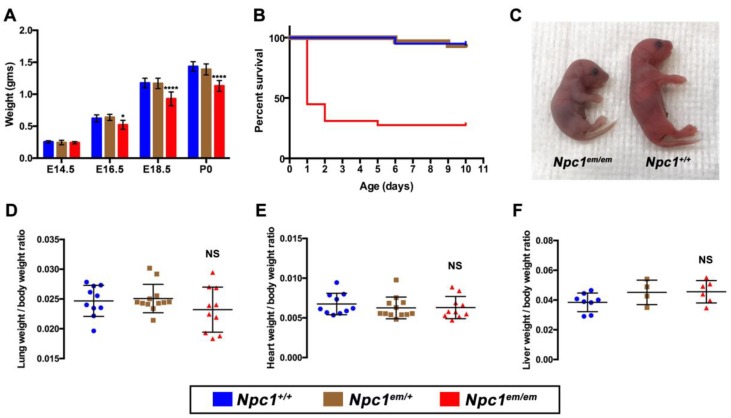
*Npc1^em/em^* mutants exhibit developmental weight reduction and neonatal lethality. (**A**) Significant growth restriction is apparent during embryogenesis in *Npc1^em/em^* mutants compared to *Npc1^+/+^* littermate controls beginning at E16.5. *Npc1^em/+^* embryos are indistinguishable from *Npc1^+/+^* mice. At E14.5, *Npc1^+/+^ n* = 4, *Npc1^em/+^ n* = 7, and *Npc1^em/em^ n* = 3. At E16.5, *Npc1^+/+^ n* = 3, *Npc1^em/+^ n* = 10, and *Npc1^em/em^ n* = 4. At E18.5, *Npc1^+/+^ n* = 6, *Npc1^em/+^ n* = 20, and *Npc1^em/em^ n* = 6. At P0/birth (following cesarean delivery), *Npc1^+/+^ n* = 16, *Npc1^em/+^ n* = 28, and *Npc1^em/em^ n* = 22. *, *p* < 0.05, ****, *p* < 0.0001, indicating significant differences between *Npc1^+/+^* and *Npc1^em/em^*. (**B**) Neonatal lethality in *Npc1^em/em^* mice. Survival plots after cesarean delivery show significant lethality of *Npc1^em/em^* mice in comparison to *Npc1^+/+^* and *Npc1^em/+^* mice (*p < 0.0001*). The majority of *Npc1^em/em^* lethality (16 out of 29 *Npc1^em/em^* mutants) occurs within 24 h after birth; all *Npc1^+/+^* and *Npc1^em/+^* littermates survive during this 24-h period. *Npc1^+/+^*, *n* = 20; *Npc1^em/+^, n* = 30; and *Npc1^em/em^*, *n* = 29. (**C**) Representative picture showing cyanosis in an *Npc1^em/em^* mutant (left) compared to an *Npc1^+/+^* littermate (right) immediately after birth. (**D**,**E**,**F**) Normalized weights (in grams) of lung, heart, and liver in *Npc1^em/em^* mutants at birth are not significantly different from *Npc1^+/+^* and *Npc1^em/+^* littermates. Wet weights of organs were analyzed at birth and normalized to total body weight; NS, not significant.

Statistical analyses were done using Prism software v6 (https://www.graphpad.com). All Chi-squared tests were performed using two degrees of freedom based on the expected number of animals per genotype. All survival analyses were done using the log-rank test (Mantel-Cox). For Figure 1A,D,E,F, one-way ANOVA tests were performed along with Bonferroni or Dunnett’s correction for multiple comparisons. 

## 3. Results

### 3.1. Reduced Homozygote Viability for Multiple Npc1 Alleles

While performing routine genotyping at postnatal day (P) 10 for mice from crosses between two *Npc1^em/+^* heterozygous mice (on a C57BL/6J inbred strain background), we consistently observed a reduced number of *Npc1^em/em^* homozygous mice. Genotyping of over 800 offspring of *Npc1^em/+^* crosses at P10 yielded only 10.5% homozygotes, a percentage significantly lower than the expected 25% (*p* < 0.0001, Table 1). Quantification of P10 offspring in two other *Npc1* mutant strains, *Npc1^m1N^* and *Npc1^I1060T^*, each on a C57BL/6J background (see Methods), also found significantly reduced percentages of homozygous *Npc1* mutants (Table 1). These results show that lethality occurs in *Npc1* homozygotes from these three mutant strains prior to P10, and are consistent with previously published reports of sub-Mendelian ratios of mutant offspring in other *Npc1* alleles, including the *Npc1^nmf164^*, *Npc1^pioneer^*, *Npc1^imagine^* and *Npc1^m1N^* alleles [30,31].

Previous studies suggested that modifiers affect phenotypic severity in both NPC1 patients and mouse models [32,34,35,36,37,38,39]. Because recent analysis of *Npc1^em/em^* mice demonstrated that strain-specific modifiers between the C57BL/6J and BALB/cJ strains had a significant effect on both lifespan and visceral pathology (Rodriguez-Gil et al., under revision), we examined neonatal lethality in these two strains. Similarly, comparison of *Npc1^em/em^* mice on C57BL/6J and BALB/cJ backgrounds showed that neonatal lethality varied by strain background, with a lower rate of lethality observed on the BALB/cJ background compared to the C57BL/6J background. We also examined neonatal lethality in mice carrying the *Npc1^m1N^* allele on these two backgrounds and observed a similar reduction in *Npc1^m1N/m1N^* lethality on the BALB/cJ background (Table S1).

### 3.2. Fetal Growth Restriction and Low Birth Weight in Npc1^em/em^ Mutants

To further characterize the developmental timeline of *Npc1^em/em^* mutant lethality, litters derived from a cross of *Npc1^em/+^* heterozygous mice were examined at embryonic day (E) 14.5, E16.5, E18.5, and P0/birth. No gross morphological defects were identified in *Npc1^em/em^* mutants from E14.5 to E18.5. However, starting at E16.5, *Npc1^em/em^* mutants showed a significant weight reduction compared to *Npc1^+/+^* and *Npc1^em/+^* littermates (Figure 1A). A distinctive small body size persisted throughout the lifespan of the *Npc1^em/em^* mutants that survived beyond the postnatal period (Rodriguez-Gil et al., under revision). 

### 3.3. Neonatal Lethality Occurs in Npc1^em/em^ Mutants

Segregation ratios were analyzed for 422 offspring from a cross of *Npc1^em/+^* heterozygous mice at E18.5–E19.5. The observed percentage of *Npc1^em/em^* homozygous mutants was not significantly different than expected (26.5%, *n* = 112/422, *p* = 0.341). This is consistent with the absence of gross morphological anomalies in *Npc1^em/em^* mutants and suggests that homozygous mutant lethality occurs between birth and P10 rather than during embryogenesis.

To examine the neonatal period in *Npc1^em/em^* mutants, we performed cesarean deliveries accompanied by maternal fostering at E19.5 (see Methods). Monitoring of these newborn pups revealed that the majority of *Npc1^em/em^* mutants (55%, *n* = 16/29) died within the first 24 h after birth, while 100% of *Npc1^+/+^* and *Npc1^em/+^* heterozygotes survived during this time period (Figure 1B). By P10, only 10% of the cesarean-delivered *Npc1^em/em^* mutants survived (*n* = 8 *Npc1^em/em^* / 79 total), a percentage that was similar to the sub-Mendelian ratios observed at P10 following natural birth (Table 1). The reduced survival of homozygous *Npc1^em/em^* mutants during the neonatal period demonstrates that *Npc1* loss of function is associated with neonatal lethality. 

### 3.4. Abnormal Lung Pathology in Npc1^em/em^ Mutants

Monitoring of newborn pups shortly after birth revealed cyanosis in *Npc1^em/em^* mutants (Figure 1C), suggesting a lack of proper oxygenation due to circulatory or respiratory dysfunction. *Npc1^em/em^* mutants also showed increased respiratory effort, evident from visible gasping and retractions. Based on this phenotypic presentation, we analyzed the organ systems that would most likely cause neonatal lethality. Analysis of histological sections found no overt morphological abnormalities in the heart, lung, or ribcage structure of *Npc1^em/em^* mutants (Figure S1A), thus ruling out heart malformations and respiratory obstruction. Specialized staining in the lung using Masson’s trichrome showed that no fibrosis was present (Figure S1B). Normalized organ weights of both lungs and heart from *Npc1^em/em^* mutants were not significantly different from the organ weights of control littermates, thus ruling out hypoplasia of these organs at birth (Figure 1D,E). Interestingly, liver histological analysis as well as liver weights of *Npc1^em/em^* mutants were similar to those of control littermates (Figure 1F), which were intriguing results given that hepatomegaly is a common NPC1 phenotype and liver failure is reported as a main cause of neonatal mortality in NPC1 patients.

**Figure 2:**
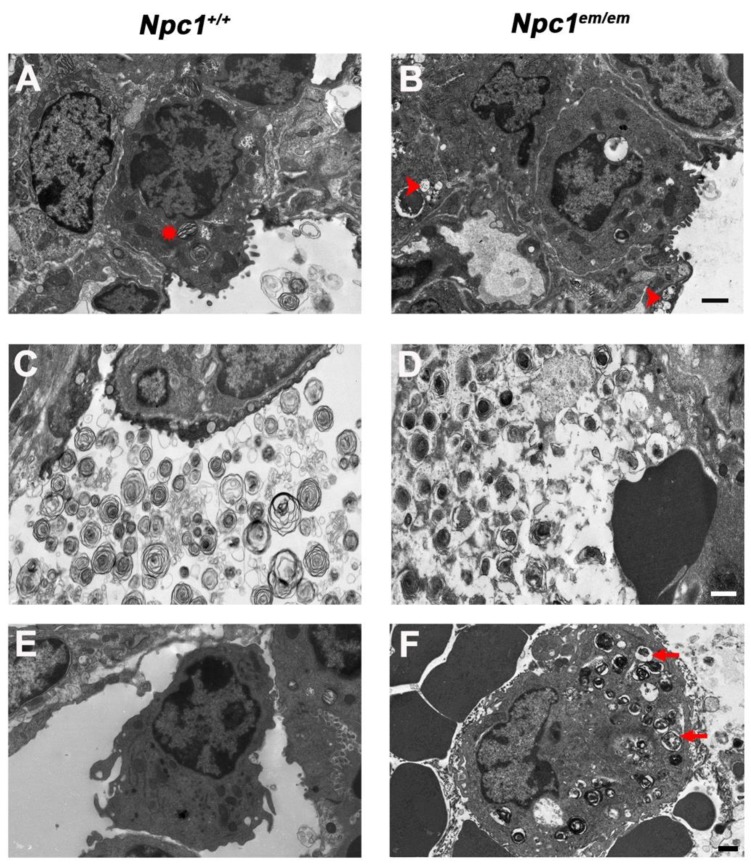
Transmission electron microscopy analysis showed that *Npc1^em/em^* newborn mutants exhibit abnormal lung pathology. (**A**,**B**) Abnormal accumulation of lipid droplets is present within the lung tissue in *Npc1^em/em^* newborn mutants (right panel). The arrowheads indicate representative lipidosis (right panel), while the asterisk indicates a normal lamellar body in a *Npc1^+/+^* type II alveolar cell (left panel), the cell type responsible for the synthesis, assembly and exocytosis of lung surfactant into the alveolar air space. (**C**,**D**) Accumulation of abnormal intra-alveolar surfactant is present in *Npc1^em/em^* newborn mutants. *Npc1^+/+^* mice (left) show lamellar body-like surfactant particles in the air space that are easily visualized by their classical “onion ring” structure. In contrast, the air space of *Npc1^em/em^* mutants (right) showed accumulation of surfactant with abnormal structures along with an amorphous extracellular material. (**E**,**F**) Foamy, enlarged alveolar macrophages filled with vacuole-like particles (arrows) are present in *Npc1^em/em^* newborn mutants (right) compared to *Npc1^+/+^* littermates (left). Macrophages were identified by their lack of microvilli and their non-polymorphic, oblong nuclei. Representative vacuole-like particles are shown by arrows. Scale bars = 1 µm.

Lung surfactant covers the surface of alveoli, acting to reduce surface tension at the air–lung interface to allow essential gas exchange [40]. Lung surfactant also plays a major role in neonatal survival [41,42]; therefore, lung tissues from *Npc1^em/em^* newborn pups were analyzed using transmission electron microscopy (TEM). This analysis showed that *Npc1^em/em^* newborn mutants contained an abnormal accumulation of lipid droplets throughout the lung tissue (Figure 2A,B). TEM analysis also revealed abnormally structured surfactant-like particles along with amorphous extracellular material accumulated in the air space of *Npc1^em/em^* mutants, which was notably different from the “onion ring” structure of normal surfactant particles present in *Npc1^+/+^* mice (Figure 2C,D). Furthermore, *Npc1^em/em^* mutants exhibited enlarged alveolar macrophages containing vacuole-like particles as a result of lipid accumulation (Figure 2E,F). Additionally, lipidosis was detected prior to birth in E16.5 *Npc1^em/em^* mutants (Figure S2), consistent with these lung abnormalities arising during embryonic development. These results indicate that *Npc1^em/em^* newborn mutants displayed labored breathing at birth accompanied by abnormal lipidosis and surfactant accumulation in the lung’s alveolar space, suggesting that neonatal lethality in *Npc1^em/em^* mutants is associated with respiratory insufficiency. 

## 4. Discussion

Model organisms that recapitulate both the heterogeneity and hallmarks of NPC1 disease are important resources that can be used to greatly improve the diagnostic assays and drug discovery that are desperately needed for this fatal disorder. The earliest presentation of NPC1 disease occurs in utero, and is followed by complications during the neonatal period and a high mortality rate within the first few weeks of life [17,18]. In this study, we discovered that mouse mutants homozygous for the *Npc1^em^* allele exhibit intrauterine growth restriction and neonatal lethality associated with abnormal lung pathology. We also provided robust quantitative data showing reduced viability prior to P10 in two other *Npc1* mutant strains, *Npc1^m1N^* and *Npc1^I1061T^*. It has been documented in previous studies as well as anecdotally that there is a reduction in the expected number of homozygous mutant animals at weaning for multiple *Npc1* alleles, but these studies did not determine the time of death [30,31]. Therefore, the perinatal lethality we describe here in *Npc1^em/em^* homozygous mice may not only be specific to this allele. Future studies will be needed to determine whether the same phenomenon causes the sub-Mendelian ratios at weaning in other *Npc1* alleles [30,31].

*Npc1^em/em^* mutants were cyanotic and displayed increased respiratory effort, suggesting a lack of proper oxygenation at birth. Recent reports have shown that severe pulmonary complications are more common in NPC1 patients with pre/perinatal onset than was previously realized [23,24]. Interestingly, the common features of clinical presentation and radiological findings in NPC1 patients with pre/perinatal onset from two recent studies indicate that these patients develop pulmonary complications that are consistent with interstitial lung disease [23,24]. This suggests that the affected lung parenchyma is unable to performed adequate gas exchange, leading to hypoxic features seen in neonatal NPC1 patients such as cyanosis, tachypnea and nail clubbing [23,24,43]. Both reports also showed relevant lung pathology featuring alveolar lipidosis and the accumulation of alveolar foam macrophages, and the images from TEM analysis of lung postmortem tissues from an affected NPC1 patient were strikingly similar to TEM images from *Npc1^em/em^* mutants. Collectively, these observations indicate that newborn *Npc1^em/em^* mutants provide a valid model to study the important pulmonary features associated with NPC1 patients with pre/perinatal onset, and they may be a useful model for discovery of therapeutic interventions for NPC1 patients suffering from respiratory complications.

The crucial role of lung surfactant in facilitating gas exchange means that surfactant plays a major role in survival at birth [42]. Mammalian surfactant exhibits a complex molecular composition of proteins and lipids, including moderate levels of sphingomyelin and abundant levels of cholesterol [44,45]. Appropriate cholesterol levels are important for surfactant stability and function, and increased amounts of cholesterol have been linked to pulmonary dysfunction [46,47]. Interestingly, NPC1 protein is localized to the limiting membrane of lamellar bodies of type II alveolar cells, and inhibition of NPC1 function has been associated with accumulation of cholesterol within the lamellar body [48]. Furthermore, abnormal surfactant composition has been reported in *Npc1* adult mice at end-stage [49], although this phenotype differs from the abnormal surfactant accumulation seen in newborn *Npc1^em/em^* mutants. Taken together, these data suggest that NPC1 dysfunction could alter the composition of pulmonary surfactant, potentially by changing cholesterol levels, thus interfering with proper alveolar gas exchange at birth. 

Foamy alveolar macrophages were present in *Npc1^em/em^* mutants at birth and have previously been shown to be present in adult *Npc1^m1N/m1N^* mice [49]. Alveolar macrophages play essential roles in the immune response as well as in processing and degrading surfactant components [45,50], and recent studies have shown that surfactant can act as an antimicrobial agent [51,52]. This is of interest because recurrent pneumonia is common in NPC1 patients, both in patients with an earlier, non-neurological presentation of NPC1 [53] and in NPC1 patients with neurological symptoms, and has been reported as the main cause of mortality in multiple patient cohorts [53,54]. Although dysphagia is common in NPC1 patients and would clearly contribute to the risk of aspiration-induced pneumonia [55], the presence of foamy alveolar macrophages as well as abnormal surfactant composition discovered in *Npc1^em/em^* mice may also be risk factors for the development of pneumonia in NPC1 disease. 

Future studies will be needed to characterize the mechanisms underlying the link between intrauterine growth restriction and loss of *Npc1*. Intrauterine growth restriction has been previously described in fetal-onset NPC1 patients [20]. One possible cause for this would be placental insufficiency, supported by the presentation of placentomegaly in multiple fetal NPC1 patients along with a report describing a fetal-onset NPC1 patient with abnormal placental pathology, including foamy cells in the villous stroma [20,22,56]. Abnormal placental changes, such as the presence of vacuolated cells, have also been associated with other lysosomal storage disorders [24]. Other important developmental pathways may also be adversely affected by *Npc1* deficiency, thus contributing to reduced body size. For example, cholesterol functions in multiple aspects of the sonic hedgehog (SHH) pathway, which regulates numerous developmental processes [57,58,59,60,61,62]. Thus, an imbalance in cholesterol homeostasis resulting from reduced NPC1 protein could have subtle effects throughout development. In support of this, abnormal expression and localization of SHH and its receptor Patched has been shown in the cerebellum of *Npc1* mutant mice at P14 as well as in NPC1 patient-derived fibroblasts [63,64]. These data along with other studies highlighting the importance of cholesterol for SHH function in the developing cerebellum suggest that important connections may exist between NPC1 and SHH signaling pathways [58,65,66]. 

Interestingly, while more than half of the *Npc1^em/em^* mutants died during the first 24 h after birth, the remaining mice survived and progressed to develop neurological symptoms beginning at 5–6 weeks of age (Rodriguez-Gil et al., under revision). The factors that allow a subset of *Npc1^em/em^* mutants to survive past 24 h of age are unknown, but it is of interest to compare these data with information on human patients with pre/perinatal-onset NPC1 disease. Approximately 8–9% of these patients develop cholestatic liver disease that ultimately leads to liver failure within a few weeks of birth [14,53]. While the presence of hepatomegaly seems to be a common clinical presentation, a subgroup of patients recovers after a few months with only transient symptoms. These patients usually only retain persistent splenomegaly or hepatomegaly until they develop NPC1-related neurological symptoms later in life [14]. The reason for this difference in clinical prognosis and survival is still poorly understood, however, the similarities between these patients and mice carrying the *Npc1^em^* allele suggest that this mouse model will be of great utility for future studies on the diagnosis and treatment of NPC1 patients at this early, critical stage of the disease.

## Figures and Tables

**Table 1:** Reduced homozygous mutant viability is observed in several *Npc1* alleles on a C57BL/6J background.

Allele	Age	Control^1^ (%)	Het (%)	Mutant (%)	Total	Chi Square *p* value^2^
*Npc1^em^*	P10	249 (29.6)	504 (59.9)	88 (10.5)	*n* = 841	*p* < 0.0001
*Npc1^m1N^*	P10	53 (32.5)	92 (56.4)	18 (11.0)	*n* = 163	*p* < 0.0001
*Npc1^I1061T^*	P10	117 (28.5)	219 (53.5)	74 (18)	*n* = 410	*p* = 0.027

***^1^*** Control = *Npc1^+/+^* littermates; ***^2^*** Compared to expected Mendelian ratios of 1:2:1; *Npc1,* Niemann-Pick disease type C1.

## Supplementary Materials

The following are available online at https://www.mdpi.com/2077-0383/9/1/12/s1, Table S1. Strain-specific differences in neonatal lethality are present in *Npc1^em^* and *Npc1^m1N^* mouse models. Figure S1: Histological analysis show no gross abnormalities or lung fibrosis in *Npc1^em/em^* mice. Figure S2: Alveolar lipidosis is present in *Npc1^em/em^* mice during embryogenesis.
