# Supplementary material for: NPC1 Deficiency in Mice is Associated with Fetal Growth Restriction, Neonatal Lethality and Abnormal Lung Pathology
Source: J Clin Med. 2019 Dec 19;9(1):12. doi: 10.3390/jcm9010012 (PMC7019814; doi:10.3390/jcm9010012)
Supplement: Supplementary file 1 [file jcm-09-00012-s001.docx]

**Table S1.** Strain-specific differences in neonatal lethality are present in *Npc1^em^* and *Npc1^m1N^* mouse models.

| **Allele** | **Strain** | **Age** | **Control^1^**  **(%)** | **Het**  **(%)** | **Mutant**  **(%)** | **Total** | **Chi Square *p* value^2^** |
| --- | --- | --- | --- | --- | --- | --- | --- |
| *Npc1^em^* | C57BL/6J | P10 | 249 (29.6%) | 504  (59.9) | 88  **(10.5)** | *n* = 841 | ***p < 0.0001*** |
|  | BALB/cJ^3^ | P10 | 41 (25.5) | 91 (56.5) | 29  **(18.0)** | *n* = 161 | ***p = 0.0016*** |
| *Npc1^m1N^* | C57BL/6J | P10 | 53 (32.5%) | 92  (56.4%) | 18  **(11.1)** | *n* = 163 | ***p < 0.0001*** |
|  | BALB/cJ | P10 | 201  (30%) | 345  (51.5%) | 123  **(18.3)** | *n* = 669 | ***p < 0.0001*** |

^1^ Control=*Npc1^+/+^* littermates; ^2^ Compared to expected Mendelian ratios of 1:2:1; ^3^ These mice were generated by backcrossing *Npc1^em^* onto BALB/cJ for 6 generations (N6) using Speed Congenic techniques (see methods).

**
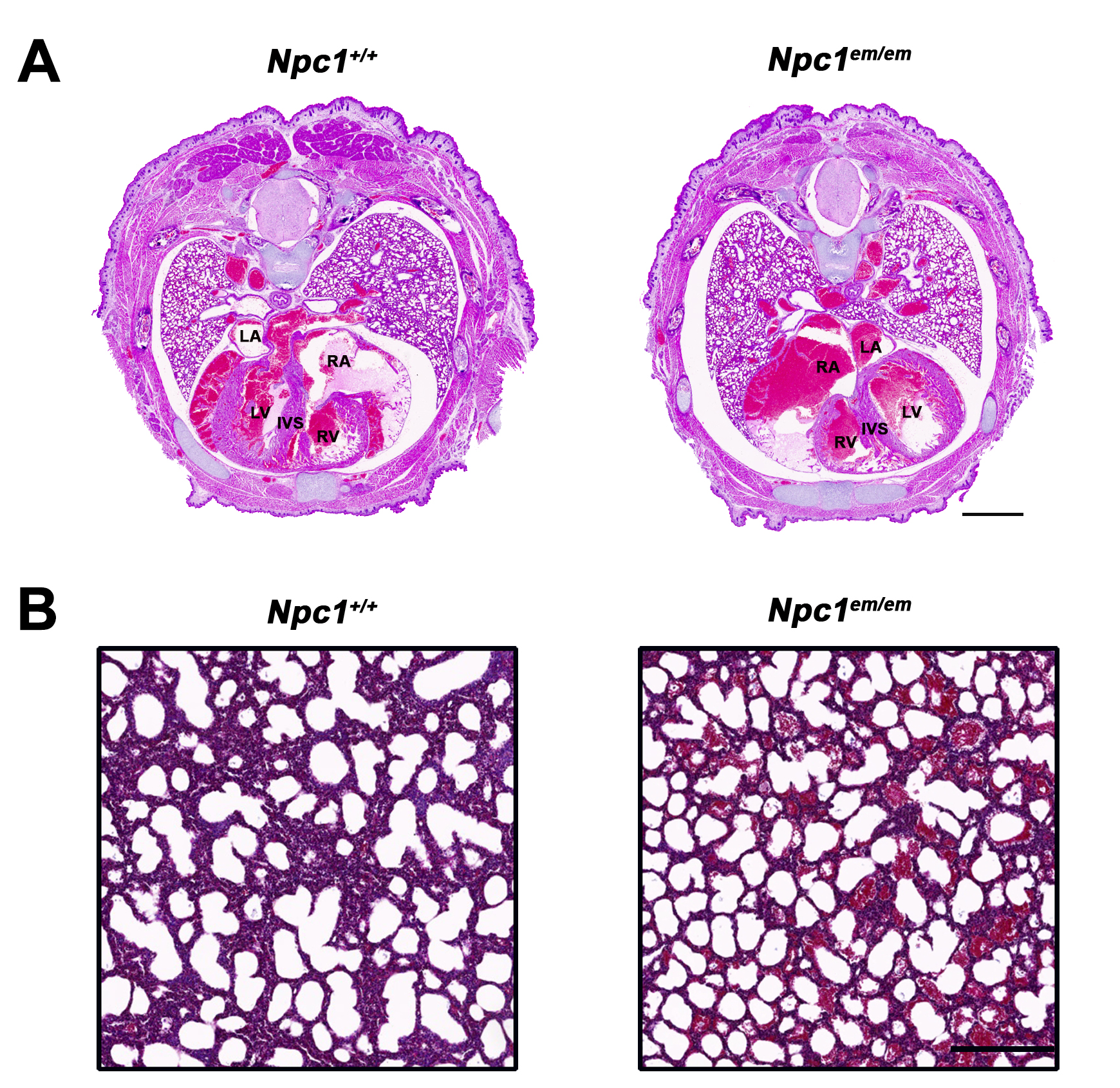
Figure S1.** Histological analyses show no gross abnormalities or lung fibrosis in *Npc1^em/em^* mice. **A)** Cross-sectional examination of the thoracic cavity from *Npc1^em/em^* mice at birth (P0). Hematoxylin and eosin staining at the cardiopulmonary level of *Npc1^+/+^* (left) and (right) mice shows normal heart morphology as well as no structural defects in the intercostal muscles. Gross lung morphology also appears normal. RA = right atrium, RV = right ventricle, IVS = interventricular septum, LA = left atrium, LV = left ventricle. Scale bar = 1mm. **B)** Histological analysis of lung tissue from *Npc1^+/+^* and *Npc1^em/em^* mice at birth. Masson’s trichrome staining shows lung fibrosis is not present. Scale bar 200um.

**
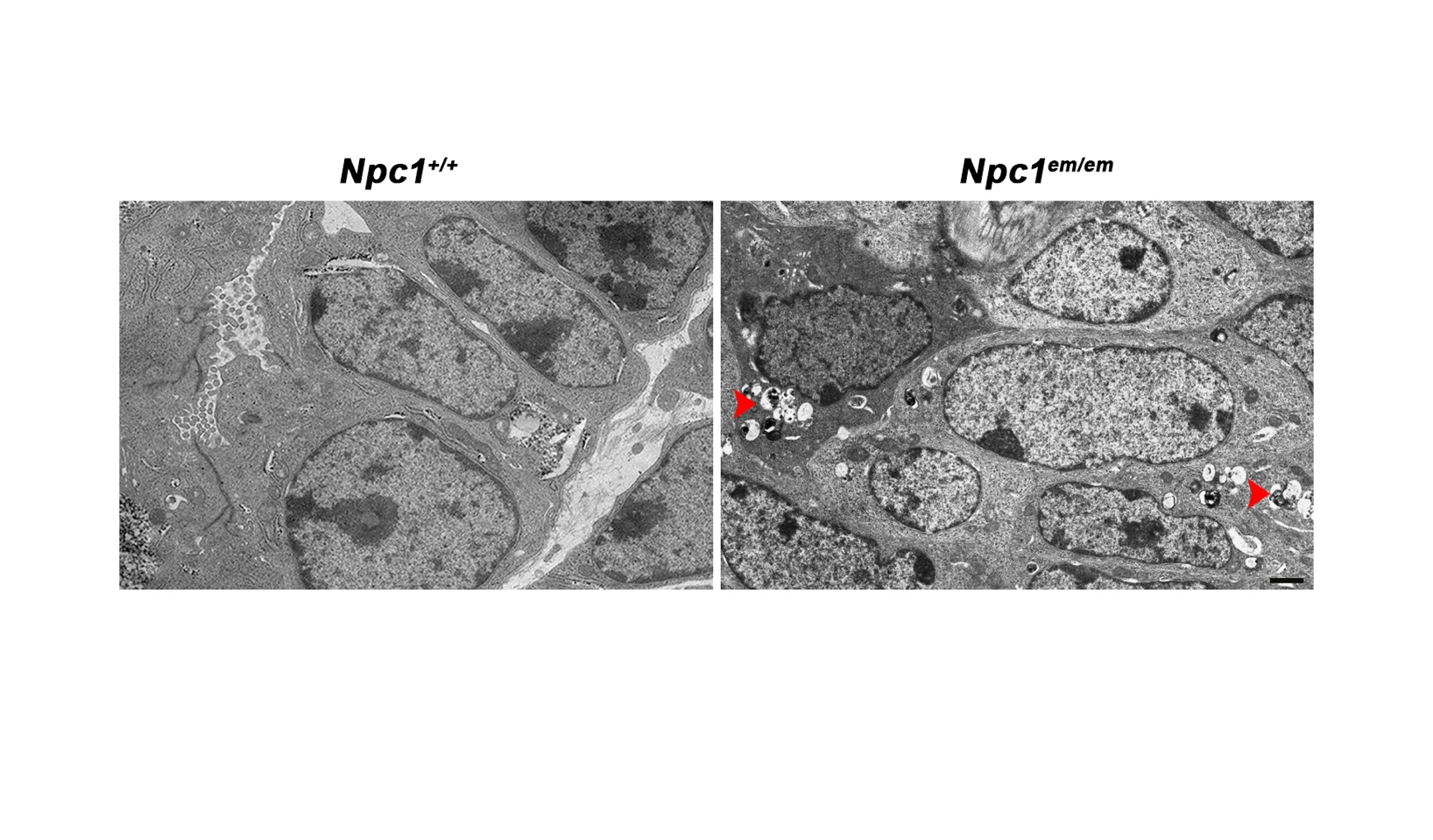
Figure S2.** Alveolar lipidosis is present in *Npc1^em/em^* mice during embryogenesis. Lipidosis (red arrowheads) within the lung tissue is seen in homozygous mutant embryos at E16.5. Lung tissue from *Npc1^+/+^* is shown on the left for comparison. Scale bar =1μm.
